# A Modified Collagen Dressing Induces Transition of Inflammatory to Reparative Phenotype of Wound Macrophages

**DOI:** 10.1038/s41598-019-49435-z

**Published:** 2019-10-04

**Authors:** Amitava Das, Motaz Abas, Nirupam Biswas, Pradipta Banerjee, Nandini Ghosh, Atul Rawat, Savita Khanna, Sashwati Roy, Chandan K. Sen

**Affiliations:** 1 Department of Surgery, IU Health Comprehensive Wound Center, Indiana Center for Regenerative Medicine and Engineering, Indiana University School of Medicine, Indianapolis, IN 46202 USA; 2 Comprehensive Wound Center and Department of Surgery, The Ohio State University Wexner Medical Center, Columbus, OH 43210 USA

**Keywords:** Inflammation, Molecular medicine

## Abstract

Collagen containing wound-care dressings are extensively used. However, the mechanism of action of these dressings remain unclear. Earlier studies utilizing a modified collagen gel (MCG) dressing demonstrated improved vascularization of ischemic wounds and better healing outcomes. Wound macrophages are pivotal in facilitating wound angiogenesis and timely healing. The current study was designed to investigate the effect of MCG on wound macrophage phenotype and function. MCG augmented recruitment of macrophage at the wound-site, attenuated pro-inflammatory and promoted anti-inflammatory macrophage polarization. Additionally, MCG increased anti-inflammatory IL-10, IL-4 and pro-angiogenic VEGF production, indicating a direct role of MCG in resolving wound inflammation and improving angiogenesis. At the wound-site, impairment in clearance of apoptotic cell bioburden enables chronic inflammation. Engulfment of apoptotic cells by macrophages (efferocytosis) resolves inflammation *via* a miR-21-PDCD4-IL-10 pathway. MCG-treated wound macrophages exhibited a significantly bolstered efferocytosis index. Such favorable outcome significantly induced miR-21 expression. MCG-mediated IL-10 production was dampened under conditions of miR-21 knockdown pointing towards miR-21 as a causative factor. Pharmacological inhibition of JNK attenuated IL-10 production by MCG, implicating miR-21-JNK pathway in MCG-mediated IL-10 production by macrophages. This work provides direct evidence demonstrating that a collagen-based wound-care dressing may influence wound macrophage function and therefore modify wound inflammation outcomes.

## Introduction

Non-resolving persistent inflammation contributes to wound chronicity^1,2^. Inflammation is required to set the process of wound healing in motion^3^. However, it is important to resolve such inflammation in a timely manner to achieve healing^4^. Wound inflammation is subject to sophisticated regulation by a number of key factors including the environment of the wound which is rich in extracellular matrix (ECM)^5^. Increased expression of matrix metalloproteinases (MMPs), enzymes that degrade ECM proteins, is a hallmark of persistent inflammation^6^. Following tissue injury and degradation of ECM, matrix fragments elicit cell signaling aimed at modulating inflammation and implementing the healing response^7^. Collagen peptides arising from ECM breakdown are known to influence the process of inflammation^8,9^.

Collagen is biodegradable and possess weak antigenic properties^10^. Collagen-based dressings are extensively used in wound care^11,12^. These dressings are biocompatible, safe and easily applicable and can be combined with other modalities of care^13^. Treatment of wounds with collagen promoted hemostasis and chemotaxis^14^. Modified collagen gel (MCG) is a bovine collagen based wound dressing. Using preclinical porcine models of excisional and ischemic wounds we have observed that MCG is effective in resolving inflammation and improving angiogenesis in these wounds^15,16^. Highly plastic wound-site macrophages play a pivotal role in tissue repair^17–20^. Depending upon micro-environmental conditions, macrophages possess a wide range of functions which are modulated through the release of several factors^21^. Successful and timely resolution of inflammation involves switching of macrophages from an inflammatory (mϕ^inf^) to a reparative (mϕ^heal^) phenotype. In the current work, we sought to evaluate the effect of MCG on macrophage function and polarization in the context of wound inflammation.

## Results

### MCG augmented recruitment of macrophage to the wound site and attenuated pro-inflammatory macrophage polarization

**Figure 1:**
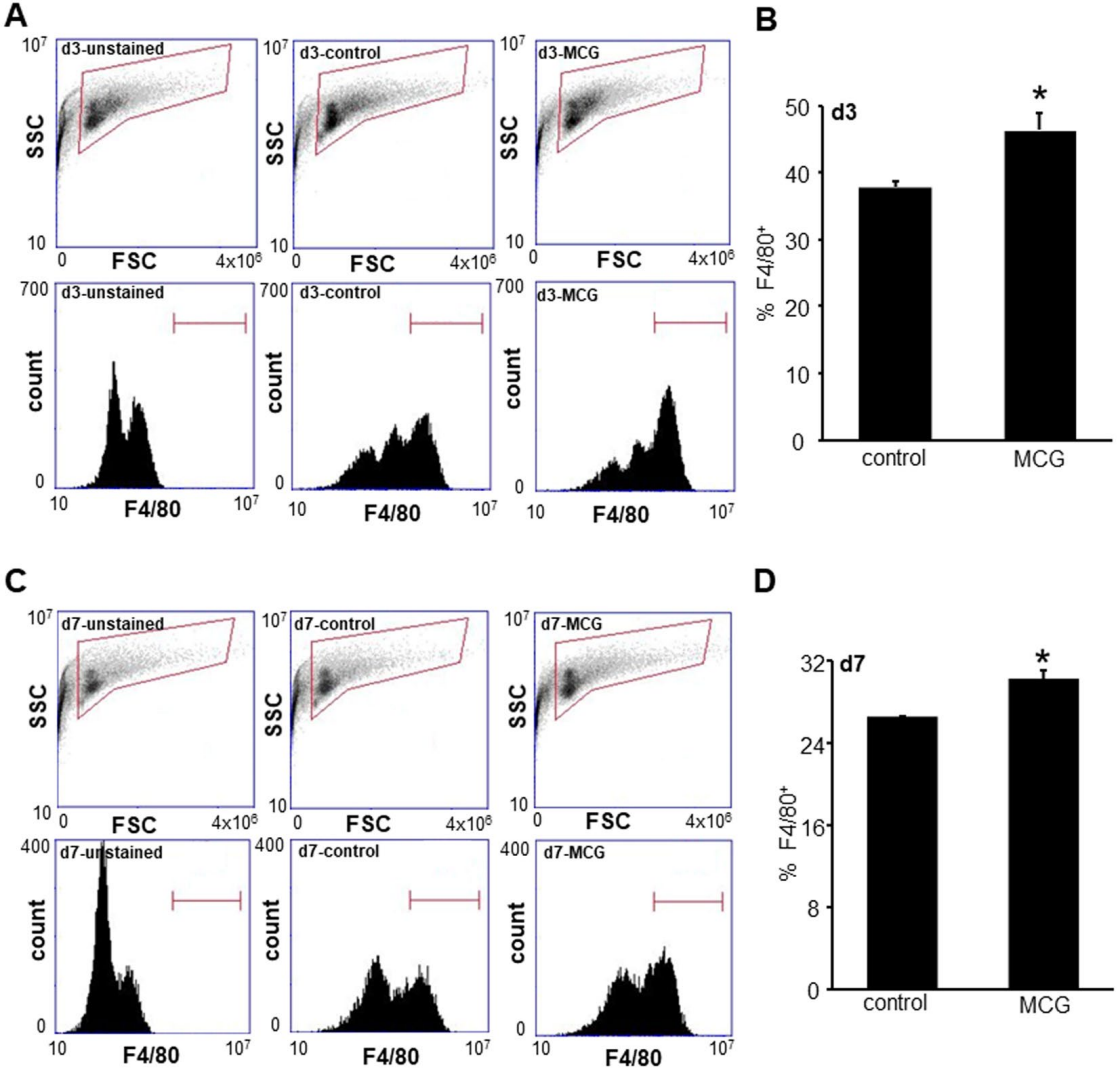
MCG increased macrophage infiltration at wound-site. Wound inflammatory cells were harvested from MCG treated PVA sponges on day 3 (**A**,**B**) and day 7 (**C**,**D**) post-implantation from C57BL/6 mice. (**A**,**C**) The cells were immunostained with F4/80 and subjected to flow cytometry analysis. (**B**,**D**) F4/80^+^ cells were quantified from the gated cell populations at both time points. Data are mean ± SEM (n = 3); *p < 0.05 compared to cells harvested from untreated PVA sponges.

**Figure 2:**
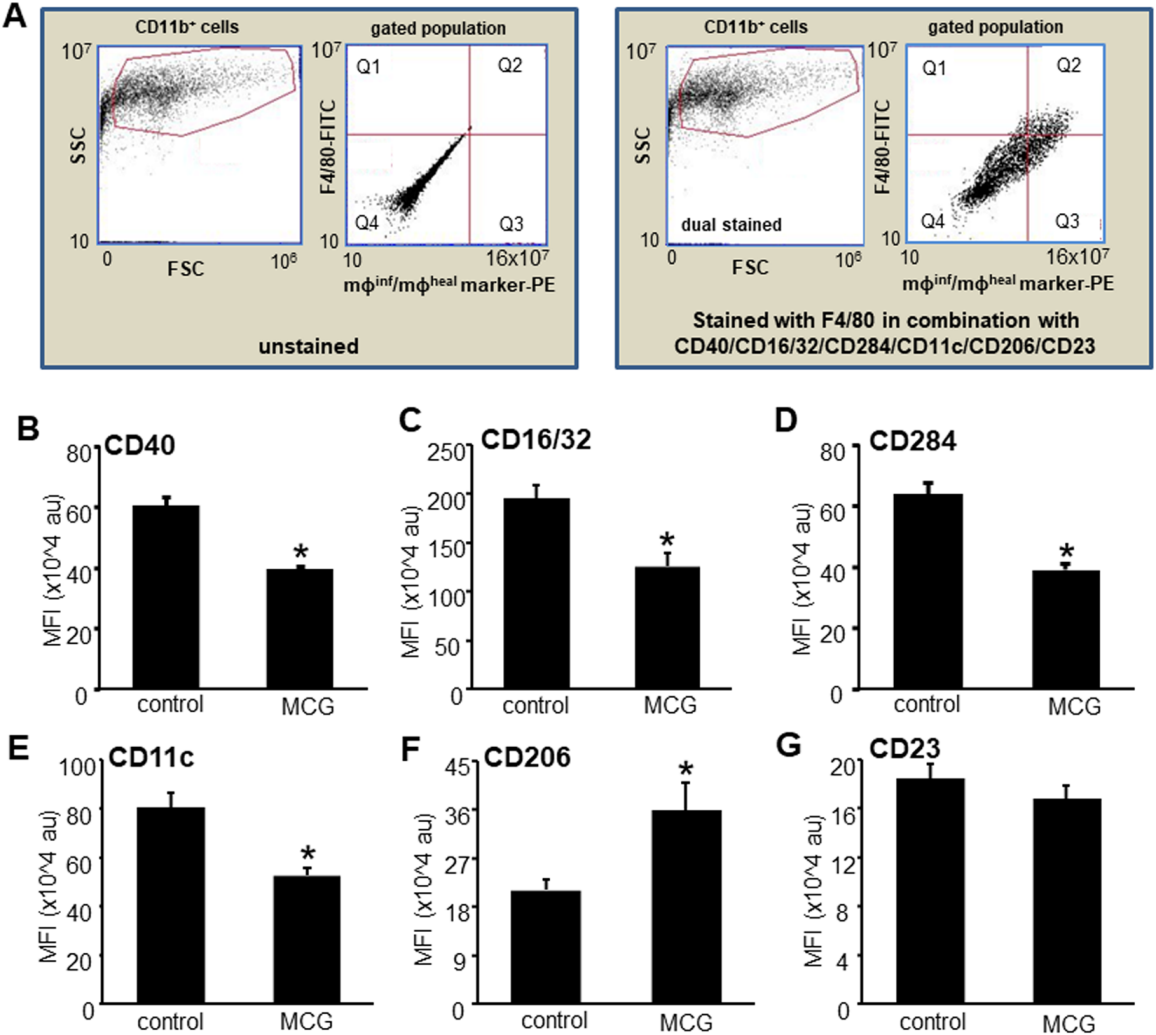
MCG attenuated mϕ^inf^ and promoted mϕ^heal^ polarization of wound macrophage in the inflammatory phase. d3 wound macrophages (CD11b^+^) were harvested from MCG treated PVA sponges subcutaneously implanted in C57BL/6 mice. The cells were immune-stained using PE conjugated mϕ^inf^/mϕ^heal^ markers and co-immunostained with FITC conjugated F4/80 and subjected to flow cytometry analysis. (**A**) Gating strategy in which the mϕ^inf^/mϕ^heal^ markers were determined in double positive cells (quadrant Q2). (**B**–**G**) Quantitative analysis of the expression (mean fluorescence intensity, MFI) is expressed as bar graphs for individual mϕ^inf^/mϕ^heal^ markers. Data are mean ± SEM (n = 6); *p < 0.05 compared to cells harvested from untreated PVA sponges.

To determine whether MCG treatment affect the macrophage abundance at the wound-site during inflammatory phases, poly vinyl alcohol (PVA) sponges soaked in MCG stock solution were implanted subcutaneously in mice. Wound inflammatory cells were harvested from PVA sponges on day 3 and day 7 post-wounding (PW), stained with FITC conjugated F4/80, a murine macrophage marker and analyzed using flow cytometry. MCG treated wound cells displayed significantly higher abundance of F4/80^+^ macrophages as compared to those from untreated wounds at both time points (Fig. 1). To determine whether MCG played a role in macrophage polarization, wound macrophages isolated from PVA sponges (CD11b^+^) were stained with F4/80-FITC and PE-conjugated mϕ^inf^/mϕ^heal^ surface markers. The double positive cells were analyzed for mϕ^inf^ surface markers CD40, CD11c, CD16/32 CD284, and mϕ^heal^ surface markers CD206 and CD23 (Fig. 2A). Significantly lower expression of all mϕ^inf^ surface markers and higher expression of mϕ^heal^ surface markers were observed in mϕ exposed to MCG on day 3 and day 7 post-implantation respectively, indicating a shift in wound macrophage polarization to an anti-inflammatory, reparative mϕ^heal^ phenotype in response to MCG (Fig. 2 and Supplementary Fig. S1).

### Upregulation of anti-inflammatory IL-10 and VEGF in MCG-treated wound cells and cultured macrophages

**Figure 3:**
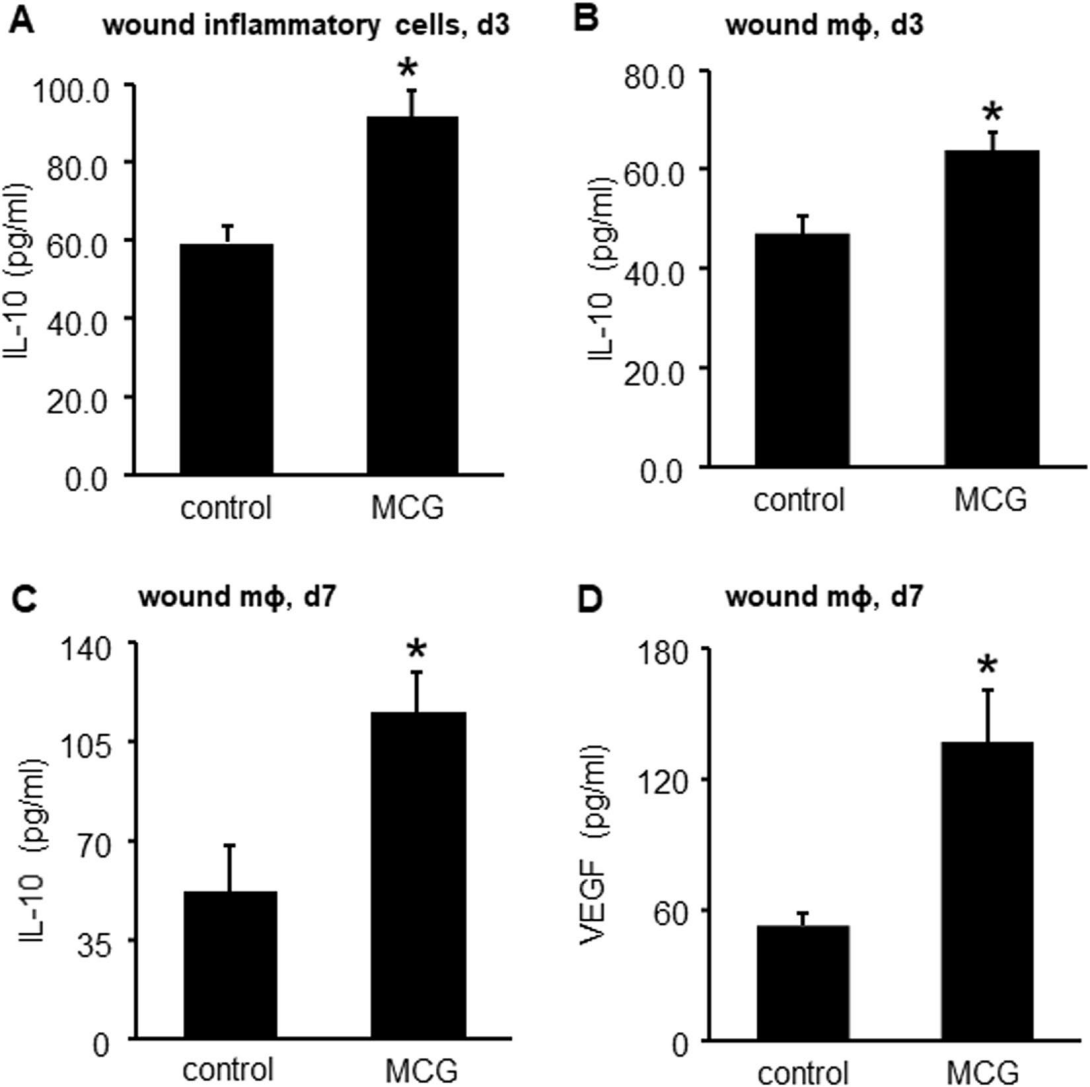
MCG induced IL-10 & VEGF release by murine wound cells. Wound inflammatory cells on d3 were harvested from MCG treated PVA sponges subcutaneously implanted in C57BL/6 mice. The wound inflammatory cells were harvested from the sponges, and subjected to ELISA for (**A**) IL-10 protein expression analysis. (**B**) d3 wound macrophages (CD11b^+^) were harvested from MCG treated PVA sponges subcutaneously implanted in C57BL/6 mice and subjected to ELISA for IL-10 protein expression. (**C**,**D**) d7 wound macrophages (CD11b^+^) were harvested from MCG treated PVA sponges subcutaneously implanted in C57BL/6 mice and subjected to ELISA for (**C**) IL-10 and (**D**) VEGF protein expression. Data are mean ± SEM (n = 5–6); *p < 0.05 compared to wound cells/macrophages harvested from untreated PVA sponges.

**Figure 4:**
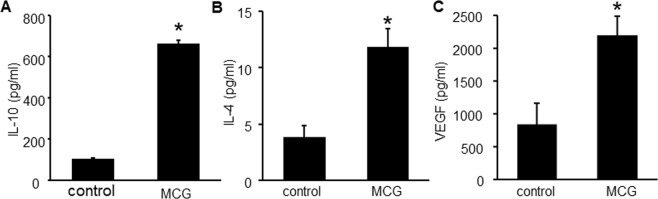
Direct MCG treatment to human cultured macrophages induces IL-10 & VEGF production. THP-1 cells were differentiated to macrophage with PMA (20 ng/ml, 48 h). The differentiated cells were then treated with MCG (100 mg/ml; 72 h) (**A**) IL-10 (**B**) IL-4 and (**C**) VEGF protein released from THP-1 differentiated human macrophages measured by ELISA. Data are mean ± SEM (n = 4–5); *p < 0.05 compared to cells harvested from untreated THP-1 cells.

Anti-inflammatory mϕ^heal^ macrophages produce copious amount of Interleukin-10 (IL-10), Interleukin-4 (IL-4) and Vascular Endothelial Growth Factor (VEGF) which helps in resolution of inflammation^22–24^ and promotes angiogenesis^25–27^. To determine whether MCG promoted an anti-inflammatory *milieu* at the wound-site, these anti-inflammatory cytokines were quantified from conditioned media of wound inflammatory cells derived from MCG-treated wounds. IL-10 protein was strongly upregulated in MCG-treated wound inflammatory cells (Fig. 3A). When studied in individual wound cell population, MCG was observed to induce IL-10 (Fig. 3B,C) and pro-angiogenic VEGF (Fig. 3D) in wound macrophages. To test a direct effect of MCG on macrophage IL-10, IL-4 and VEGF production, wound macrophages and differentiated THP-1 derived macrophages were utilized. Measurement of protein by ELISA demonstrated significant induction of IL-10, IL-4 and VEGF protein following treatment with MCG in both wound macrophages and differentiated THP-1 cells (Supplementary Fig. S2A–C and Fig. 4).

### MCG induced the efferocytosis-JNK-miR-21 pathway

**Figure 5:**
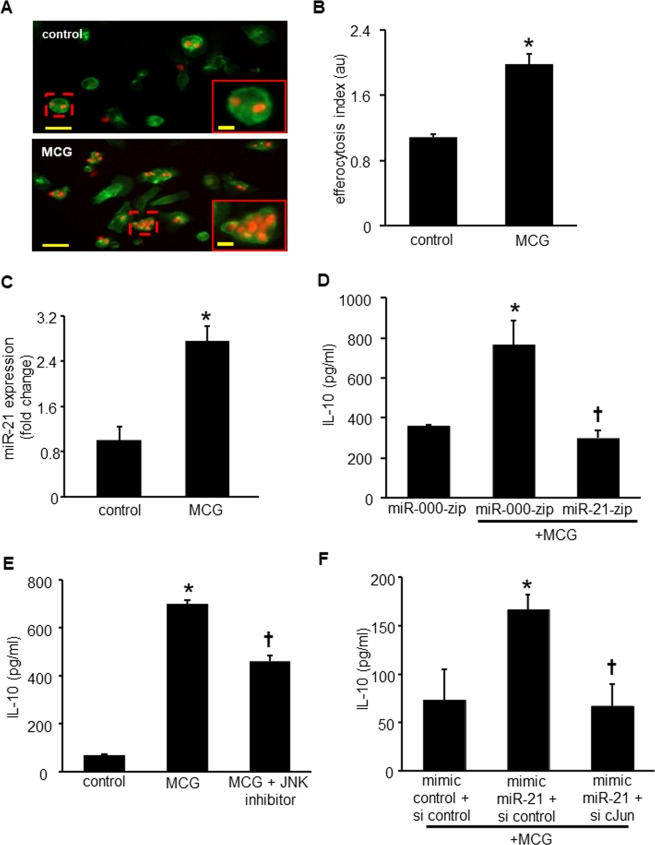
MCG promotes macrophage anti-inflammatory phenotype *via* promoting efferocytosis-JNK-miR-21 pathway. (**A**) PVA sponges were treated with MCG (2.5 g/ml), implanted subcutaneously in C57BL/6 mice. Day 3 wound cells were harvested from the sponges and subjected to efferocytosis assay. Representative images showing harvested MCG-treated macrophages (green, F4/80) cultured with apoptotic thymocytes (red, CMTMR cell tracker). (**B**) Efferocytosis index of apoptotic thymocytes engulfed by macrophages, calculated as total number of apoptotic cells engulfed by macrophages in a field of view divided by total number of macrophage present in the same field of view. Data are mean ± SEM (n = 4); *p < 0.05 compared to control. (**C**) miR-21 expression in mouse inflammatory cells collected from MCG-treated sponges at day 3 post-implantation. Data are mean ± SEM (n = 4); *p < 0.05 com*p*ared to control. (**D**) IL-10 production in miR-21-zip cells after treatment with MCG(100 mg/ml). Data are mean ± SEM (n = 3–5); *p < 0.05 compared with MCG untreated miR-000-zip (control) cells; ^†^*p* < 0.05 compared with MCG treated miR-000–zip cells. (**E**) IL-10 production in differentiated THP-1 cells after treatment with pharmacological JNK inhibitor (420119 JNK Inhibitor II, 20 µM) and MCG (100 mg/ml). Data are mean ± SEM (n = 4); *p < 0.05 compared with MCG untreated (control) cells; ^†^*p* < 0.05 compared with MCG-treated and JNK inhibitor untreated cells. (**F**) IL-10 production in THP-1 cells transfected with mimic miR-21 and si-cJun followed by treatment with MCG. Data are mean ± SEM (n = 5); *p < 0.05 compared with mimic control + si control transfected cells; ^†^*p* < 0.05 compared with mimic miR-21 + si control transfected cells.

**Figure 6:**
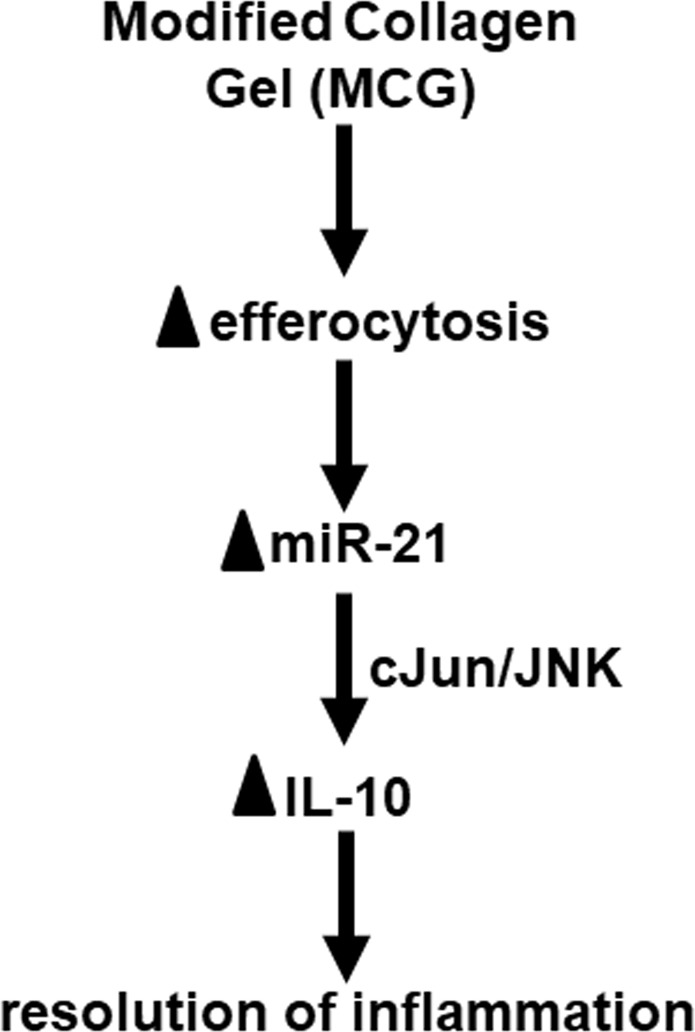
Proposed mechanism of action of Modified Collagen Gel-induced resolution of inflammation.

We have reported that engulfment of apoptotic cells by macrophages (*aka*, efferocytosis) is a signaling cue that drives polarization to anti-inflammatory mϕ^heal^ phenotype *via* miR-21- programmed cell death 4 (PDCD4)-IL-10 pathway^18^. Thus, the effect of MCG treatment on macrophage efferocytosis activity was determined. A significantly elevated efferocytosis index was noted in macrophages treated with MCG as compared to matched untreated controls (Fig. 5A,B). Successful efferocytosis is known to induce miR-21 expression, which *via* phosphatase and tensin homolog (PTEN) and PDCD4 silencing, switches macrophage to an anti-inflammatory mϕ^heal^ phenotype^18^. In this work, MCG-induced efferocytosis was associated with elevated miR-21 expression (Fig. 5C). Interestingly, MCG-induced IL-10 expression was blunted under conditions of miR-21 knockdown (Fig. 5D, Supplementary Fig. S3A). This line of evidence recognizes miR-21 as a mechanism implicated in MCG-induced IL-10 production by macrophages. We have previously reported that pharmacological inhibition of c-Jun N-terminal kinase (JNK) or knockdown of cellular c-Jun resulted in significant downregulation of inducible IL-10 protein expression, demonstrating a direct role of c-Jun and JNK in LPS-induced IL-10 expression in human monocyte-derived macrophages^18^. The JNK inhibitor (420119 JNK Inhibitor II) significantly inhibited MCG-induced IL-10 production (Fig. 5E). To further determine if MCG → miR-21 → IL-10 induction is *via* JNK pathway, THP-1 cells were transfected with miRIDIAN hsa–miR-21 mimic to increase cellular miR-21 abundance (Supplementary Fig. S3B) followed by knockdown of c-Jun using siRNA(Supplementary Fig. S3C) and treatment with MCG. Knocking down c-Jun under these conditions resulted in abrogation of MCG-induced IL-10 even in high miR-21 conditions suggesting a central role of cJun-JNK pathway in MCG → miR21 induced IL-10 production (Fig. 5F). Finally, a summary of the proposed pathway implicated in anti-inflammatory effect of MCG *via* IL-10 production has been presented (Fig. 6).

## Discussion

The microenvironment of tissue injury is characterized by the infiltration of visiting immune cells in a situation where collagen and its fragmented products are in abundance. Collagen and its degradation products have been recognized to induce signal transduction which in turn modulates several physiological functions like cell adhesion and migration, hemostasis and immune function^28,29^. Interestingly, a clostridial collagenase ointment resolves inflammation through a PGE_2_-EP_4_-STAT6 signaling pathway^30^. Collagen degraded to its peptide components is readily phagocytosed by macrophages at the wound-site^31,32^. Whether such engulfment of collagen peptides induces any cellular signaling in macrophages remains unknown. This report provides maiden evidence demonstrating that a modified collagen based wound dressing (MCG), generated by tryptic digestion and composed of short and long chain peptides of collagen, induces mϕ^heal^-like polarization in wound macrophages, including production of copious amounts of anti-inflammatory and pro-angiogenic response by these cells.

Circulating monocytes recruited to the wound tissue differentiate to macrophages that are critical in orchestrating the inflammatory and subsequent repair process at the wound-site^33–35^. Consistent with findings of the current study, an increased macrophage infiltration in excisional wounds treated with modified collagen gel in a porcine model was also noted suggesting that MCG possess a macrophage chemoattractant property^16^. LC-MS/MS studies from our laboratory have demonstrated that MCG is composed of long and short chain peptides derived from collagen^16^. Synthetic polypeptides such as pentameric (Pro-Pro-Gly)_5_ form as well as peptides of bovine collagen digested by collagenase are known to display potent chemoattractant activity for rat alveolar macrophages^36^ and human monocytes^37^. The exact mechanism of collagen peptide mediated macrophage chemoattractant function remains unclear. It is plausible that collagen peptides promote production of Monocyte Chemoattractant Protein-1 (MCP-1), a potent macrophage chemo-attractant, thereby increasing the macrophage infiltration.

Once extravasated, macrophage phenotype and function is guided by environmental cues^18,38^. At the wound-site, macrophages are known to serve functionally distinct roles including the classical (proinflammatory, mϕ^inf^) and alternative (anti-inflammatory, prohealing, mϕ^heal^) activation states^35,38–40^. While the pro-inflammatory mϕ^inf^ macrophages are responsible for the clearing of infectious agents, the mϕ^heal^ macrophages are more reparative in nature and enable timely resolution of inflammation and promote angiogenesis^15,34,41^. Chronic diabetic ulcers with unresolved inflammation display aberrant mϕ^inf^:mϕ^heal^ macrophage ratio and an imbalance between pro- and anti-inflammatory environment^42,43^. CD40, CD16-32, CD11c and CD284 (TLR4) are well-established markers of mϕ^inf^ macrophage polarization^34,39,40,44,45^. Functional wound macrophages treated with MCG *in vivo* displayed a decrease in mϕ^inf^ macrophage polarization at the inflammatory phases indicative of a shift in the wound macrophage polarization from mϕ^inf^ to mϕ^heal^. IL-10, also known as human cytokine synthesis inhibitory factor (CSIF)^46^, is a cytokine with anti-inflammatory properties^47–49^ while VEGF is a potent angiogenic factor^50^. MCG-induced shift in the phenotype of the wound macrophages was coupled with induction of IL-10 and VEGF. These findings are consistent with increased IL-10, Mannose Receptor C-Type 1 (Mrc-1) and C-C Motif Chemokine Receptor 2 (CCR2) expression in MCG-treated wounds as previously reported in a porcine model^15^. Following MCG treatment, increased mϕ^heal^ macrophage polarization was associated with an increased wound angiogenesis^15,16^. Given that anti-inflammatory tissue mϕ have been directly implicated in angiogenesis^27,51^, it is plausible that the MCG-induced mϕ^heal^ polarization of macrophages promoted wound angiogenesis^15^.

Mechanism of macrophage polarization includes complex interplay of multiple signaling pathways and transcription factors^52^. This work identified miR-21 as a major driver of MCG-induced macrophage polarization. Although sample size was modest, the findings were robust primarily because of consistent effect. An overload of apoptotic cells at the wound-site causes inflammation to persist^53^. We have recently underscored a major role of efferocytosis and microRNA-21 (miR-21) in macrophage transition from mϕ^inf^ to an anti-inflammatory mϕ^heal^ phenotype featuring increased IL-10^18^. Efferocytosis or successful engulfment of apoptotic cells is known to promote an anti-inflammatory response in macrophages^54,55^ including induction of miR-21 expression^18^. An impairment of efferocytosis in diabetic wounds led to unresolved inflammation^19,53^. miR-21 promoted anti-inflammatory mϕ^heal^ like response in human macrophages by directly targeting phosphatase and tensin homolog (PTEN) and programmed cell death protein 4 (PDCD4) that subsequently inhibited NF-κB → TNF-α or promoted JNK → AP-1 → IL-10 production^18^. Blocking of JNK resulted in an attenuation of MCG-induced IL-10 production suggesting that the anti-inflammatory effects of MCG involves miR-21 targeting PDCD4 followed by activation of JNK → AP-1 → IL-10 pathway^18^.

Collagen based wound dressings have been widely used in effective treatment of chronic wounds^11,56^. Recent studies from our laboratory have reported improved wound macrophage function and epithelialization using another stabilized collagen matrix dressing^12^. The current understanding of the mechanisms of action of these dressings include (i) serving as a substrate for high matrix metalloproteinase (MMP) in chronic wound environment; (ii) the chemotactic property of the collagen breakdown products for cells critical in formation of granulation tissue and (iii) exudate management as a result of the high absorptive property. This work identified and characterized a novel mechanism of action of collagen based wound dressings in modifying wound macrophage inflammatory response. MCG promoted an anti-inflammatory proangiogenic mϕ^heal^-like macrophage phenotype *via* miR-21-cJun/JNK mediated signaling pathway. Results of this study were obtained from the study of macrophages that were activated *in vivo* employing a standardized PVA sponge implantation model^19,30,53,57^. The reported observations provide a valuable paradigm that is ready to be tested on macrophages directly isolated from the chronic wound patients. The findings of this work add a new dimension to our understanding of macrophage-ECM interactions. It provides firm mechanistic explanation addressing the significance of collagen based wound-care dressings.

## Materials and Methods

### Polyvinyl alcohol (PVA) sponge implantation model

The animal studies were approved by, and all methods were performed in accordance with the relevant guidelines and regulations set by The Ohio State University’s Institutional Animal Care and Use Committee and Indiana University’s Institutional Animal Care and Use Committee. Circular sterile PVA sponges (8 mm diameter) were subcutaneously implanted on the back of 8-12-week old C57BL/6 mice under anesthesia induced by isoflurane inhalation as previously described^19,30,53,57^. PVA sponges were soaked in MCG stock solution (2.5 g/ml) for overnight (16 h). Soaking PVA sponges (each sponge of volume ~100 mm^3^) resulted in each sponge getting coated with ~125 mg of MCG solution. The weight of sponges post-soaking was measured to ensure equal loading of the MCG solution to each sponge. PVA sponges containing either MCG or saline (control) were then subcutaneously inserted into each animal. MCG was obtained as Stimulen^TM^ gel from Southwest Technologies Inc. (North Kansas City, MO)^15,16^. Harvesting of the PVA sponges were done on day 3/day 7 post-implantation following euthanasia. All sponges were removed and placed in sterile saline. Repeated compression of the sponges in saline resulted in a wound cell suspension which was then filtered with a 70 µm nylon cell strainer to eliminate all debris, followed by hypotonic lysis with ice cold deionized water to remove the red blood cells^19,53,57^. Wound macrophages (CD11b^+^) were obtained from day 3/day 7 wound cell infiltrate by magnetic bead based sorting as previously described^19,53^.

### Immunostaining and flow cytometry

Markers used to determine monocyte and/or macrophage subsets comprised: FITC-F4/80 (Serotec) and PE-CD16/32, PE-CD11c, PE-CD40, PE-CD284, PE-CD206, PE-CD23(eBioscience). The cells were stained with surface markers and recorded using BD Accuri flow cytometry (BD Biosciences) and analyzed as previously described^17,30,58,59^.

### Cell culture, differentiation, and treatment

The human THP-1 monocytic cell-line was cultured and differentiated to macrophages using PMA treatment as previously described^18,57^. The macrophages were treated with MCG as described previously^16^.

### RNA extraction, reverse transcription and quantitative real time-PCR (qRT-PCR)

Total RNA was isolated from the cells using the mirVana RNA Isolation Kit (Ambion, Austin, TX), according the manufacturer’s directions as previously described^15,16,18,19,53,57^. miRNA expression was determined using specific Taqman assays and miRNA RT Kit (Applied Biosystems, Foster City, CA)^15,16,18,19,53,57,60^.

### Enzyme-linked immunosorbent assay (ELISA)

For the assay, wound cells or wound mϕ (isolated from subcutaneously implanted PVA sponges) or THP-1 cells were seeded in 24-well plates cells (equally seeded for control and treatment). Cytokine levels were measured from commercially available ELISA kits as previously described^12,18,19,30,53,57^ and are expressed as per million cells.

### Apoptotic cell clearance (efferocytosis) assay

Mouse macrophages that infiltrated PVA sponges were isolated as previously described^19,53^ and seeded into 8-chambered slides. Apoptosis was induced in mouse thymocytes and the apoptotic cells were used to perform the efferocytosis assay as previously described^19,53^.

### THP-1 cells with stable knockdown of miR-21

Stable knockdown of miR-21 was achieved in THP-1 cells using lenti-miR-000-zip (control) or lenti-miR-21-zip vectors and puromycin selection as previously described^18^ followed by treatment with PMA to differentiate into macrophages^18,57^.

### miRIDIAN miR mimic and small interfering RNA delivery

Delivery of miRNA mimic and small interfering (si) RNA were be performed as previously described^18,61^.

### Immunocytochemistry and imaging

Cytospin smears from THP-1 cells suspension were fixed in cell fixation buffer (BD Cytofix, BD Biosciences, CA) for 10 min. Following fixation, cells were washed, blocked in 10% normal goat serum (NGS) for 30 min and were incubated in primary antibodies for cJun (1:100; Abcam). Fluorescence tagged secondary antibody detection was performed with Alexa Fluor 488 secondary antibody (1:200, Life Technologies) as described previously^30^. Fluorescent images were collected using collected using confocal microscopy (LSM880). Image analysis was performed using Zen (Zeiss) software to quantitate fluorescence intensity.

### Statistical analysis

Data are reported as mean ± SEM of 3–6 experiments as shown in the figure legends. Student’s t-test (two-tailed) and ANOVA were applied to determine the significance. *p* < 0.05 was considered to be statistically significant.

## Supplementary information

Supplementary dataset 1
