# Supplementary material for: A Modified Collagen Dressing Induces Transition of Inflammatory to Reparative Phenotype of Wound Macrophages
Source: Sci Rep. 2019 Oct 4;9:14293. doi: 10.1038/s41598-019-49435-z (PMC6778115; doi:10.1038/s41598-019-49435-z)
Supplement: Supplementary file 1 — Supplementary dataset 1 [file 41598_2019_49435_MOESM1_ESM.pdf]

A Modified Collagen Dressing Induces Transition of Inflammatory to Reparative Phenotype of Wound Macrophages

Amitava Das[1,2]*, Motaz Abas[2]*, Nirupam Biswas[1,2], Pradipta Banerjee[1,2], Nandini Ghosh[1,2], Atul Rawat[1], Savita Khanna[1,2], Sashwati Roy[1,2] and Chandan K. Sen[1,2]

[1]Department of Surgery, IU Health Comprehensive Wound Center, Indiana Center for Regenerative Medicine and Engineering, Indiana University School of Medicine, Indianapolis, IN 46202. [2]Comprehensive Wound Center and Department of Surgery, The Ohio State University Wexner Medical Center, Columbus, OH, 43210.

*Contributed equally to this work

Running Title: Collagen gel on wound macrophage function

Address correspondence to:

Chandan K. Sen, PhD

975 W Walnut St, Suite 454

Medical Research Library Building,

Indiana University School of Medicine

Indianapolis, IN 46202.

Tel. 317 278 2736

E-mail: cksen@iu.edu

Supplementary Figure Legends

Figure S1. MCG promotes mϕ^{heal} polarization of wound macrophage in the healing phase. D7 wound macrophages ($CD11b^+$) were harvested from MCG treated PVA sponges subcutaneously implanted in C57BL/6 mice. The cells were immune-stained using PE conjugated mϕ^{inf}/mϕ^{heal} markers and co-immunostained with FITC conjugated F4/80 and subjected to flow cytometry analysis. **(A-F)** Quantitative analysis of the expression (mean fluorescence intensity, MFI) is expressed as bar graphs for individual mϕ^{inf}/mϕ^{heal} markers. Data are mean ± SEM (n = 6); *$p < 0.05$ compared to macrophages harvested from untreated PVA sponges.

Figure S2. MCG induced IL-10, IL-4 & VEGF release by murine wound macrophages. (**A-C**) d7 wound macrophages ($CD11b^+$) were treated with MCG *ex vivo* (100 mg/ml; 24h) and subjected to ELISA for **(A)** IL-10, **(B)** IL-4 and **(C)** VEGF. Data are mean ± SEM (n = 3-6); *$p < 0.05$ compared to untreated macrophages.

Figure S3. (A) miR-21 expression in miR-000–zip or miR-21–zip cells. Data are mean ± SEM (n = 4); *$p < 0.05$ compared with miR-000–zip cells. **(B)** miR-21 expression in miRIDIAN hsa–miR-21 mimic or control mimic transfected cells. Data are mean ± SEM (n = 4); *$p < 0.05$ compared with macrophages transfected with control mimic. **(C)** Fluorescence microscopy images of c-Jun protein expression in THP-1 following knockdown of si cJun. Counterstaining was performed using DAPI (blue, nuclear). Bar graph presents quantitation of cJun. Data are mean ± SEM (n = 5-6); *$p < 0.05$.

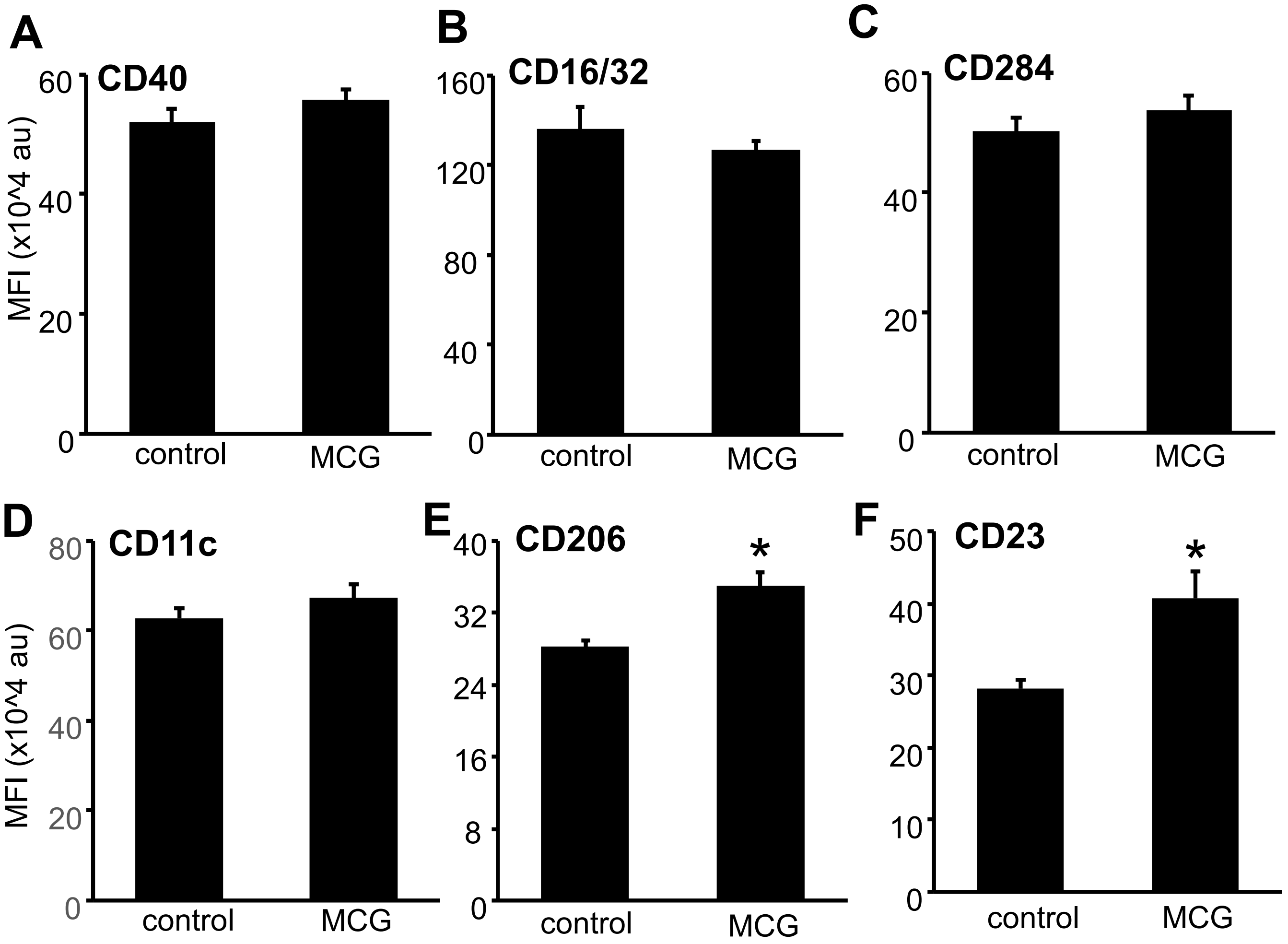
A
CD40
60
40
20
0
MFI (x10^4 au)
control
MCG
B
CD16/32
160
120
80
40
0
control
MCG
C
CD284
60
40
20
0
control
MCG
D
CD11c
80
60
40
20
0
MFI (x10^4 au)
control
MCG
E
CD206
*
40
32
24
16
8
0
control
MCG
F
CD23
*
50
40
30
20
10
0
control
MCG

Figure S1

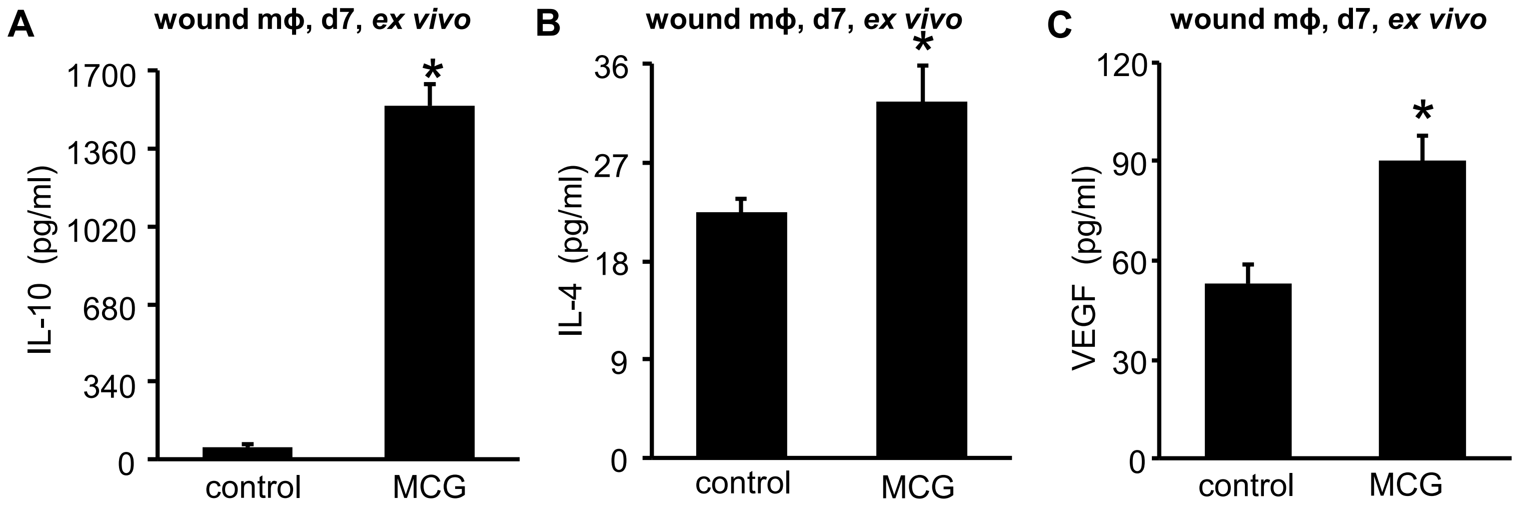
A
wound mϕ, d7, ex vivo
1700
1360
1020
680
340
0
IL-10 (pg/ml)
*
control
MCG
B
wound mϕ, d7, ex vivo
36
27
18
9
0
IL-4 (pg/ml)
*
control
MCG
C
wound mϕ, d7, ex vivo
120
90
60
30
0
VEGF (pg/ml)
*
control
MCG

Figure S2

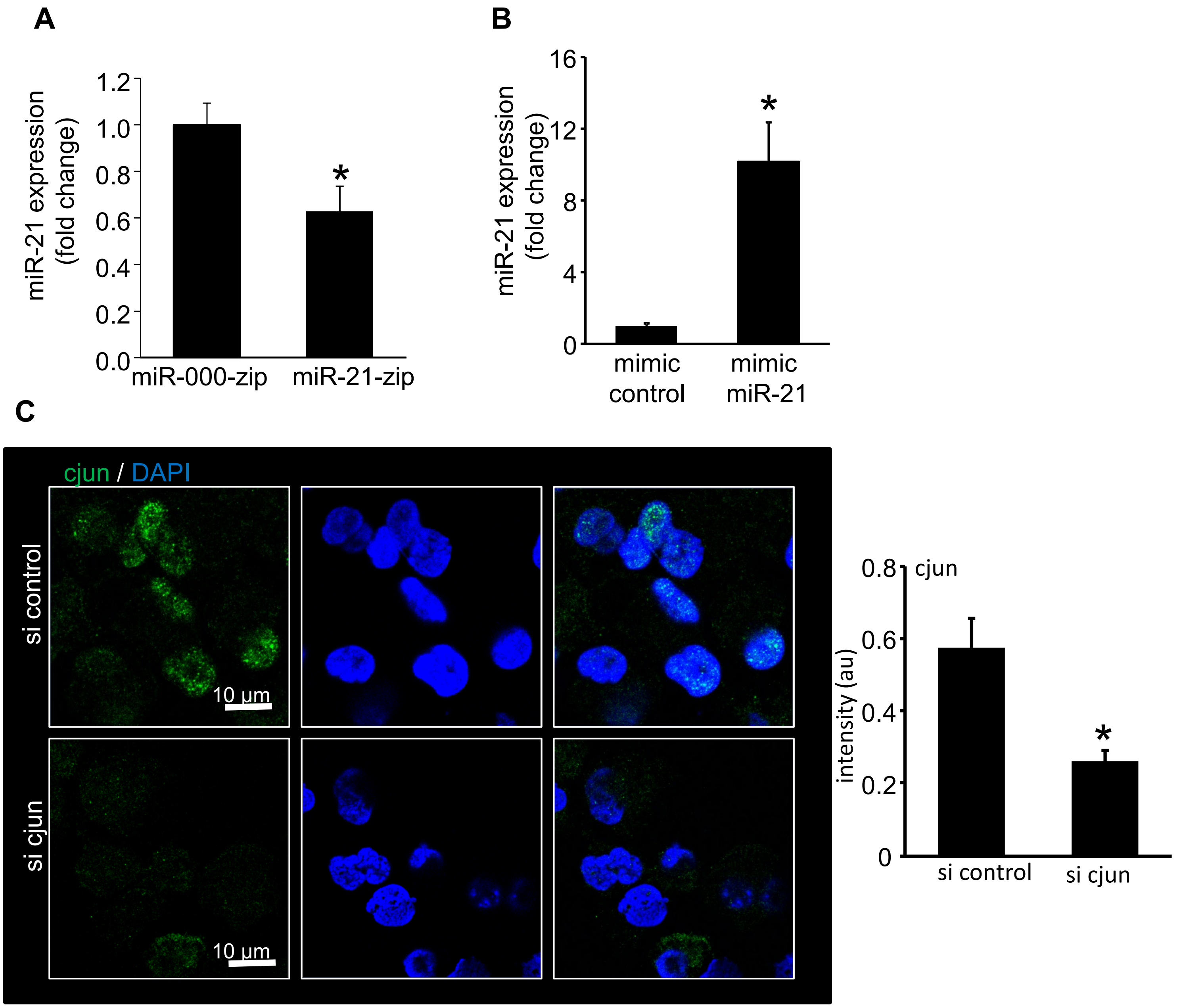

A
miR-21 expression (fold change)
1.2
1.0
0.8
0.6
0.4
0.2
0.0
*
miR-000-zip
miR-21-zip
B
miR-21 expression (fold change)
16
12
8
4
0
*
mimic control
mimic miR-21
C
cjun / DAPI
si control
si cjun
10 µm
10 µm
cjun
intensity (au)
0.8
0.6
0.4
0.2
0
*
si control
si cjun

Figure S3
